# Cross-crop resistance of *Spodoptera frugiperda* selected on *Bt* maize to genetically-modified soybean expressing Cry1Ac and Cry1F proteins in Brazil

**DOI:** 10.1038/s41598-020-67339-1

**Published:** 2020-06-22

**Authors:** Eduardo P. Machado, Gerson L. dos S. Rodrigues Junior, Fábio M. Führ, Stefan L. Zago, Luiz H. Marques, Antonio C. Santos, Timothy Nowatzki, Mark L. Dahmer, Celso Omoto, Oderlei Bernardi

**Affiliations:** 1 Department of Plant Protection, Federal University of Santa Maria (UFSM), Roraima Avenue 1000, Santa Maria, Rio Grande do Sul 97105-900 Brazil; 2 Corteva Agriscience, Alameda Itapecuru, 506, Alphaville, Barueri, SP 06454-080 Brazil; 3 Corteva Agriscience, 7000NW 62nd Ave, Johnston, IA 50131 USA; 4 Department of Entomology and Acarology, Luiz de Queiroz College of Agriculture (ESALQ), University of São Paulo (USP), Pádua Dias avenue 11, Piracicaba, São Paulo 13418-900 Brazil

**Keywords:** Entomology, Zoology

## Abstract

*Spodoptera frugiperda* is one of the main pests of maize and cotton in Brazil and has increased its occurrence on soybean. Field-evolved resistance of this species to Cry1 *Bacillus thuringiensis* (*Bt*) proteins expressed in maize has been characterized in Brazil, Argentina, Puerto Rico and southeastern U.S. Here, we conducted studies to evaluate the survival and development of *S. frugiperda* strains that are susceptible, selected for resistance to *Bt*-maize single (Cry1F) or pyramided (Cry1F/Cry1A.105/Cry2Ab2) events and F_1_ hybrids of the selected and susceptible strains (heterozygotes) on DAS-444Ø6-6 × DAS-81419-2 soybean with tolerance to 2,4-d, glyphosate and ammonium glufosinate herbicides (event DAS-444Ø6-6) and insect-resistant due to expression of Cry1Ac and Cry1F *Bt* proteins (event DAS-81419-2). Susceptible insects of *S. frugiperda* did not survive on Cry1Ac/Cry1F-soybean. However, homozygous-resistant and heterozygous insects were able to survive and emerge as fertile adults when fed on Cry1Ac/Cry1F-soybean, suggesting that the resistance is partially recessive. Life history studies revealed that homozygous-resistant insects had similar development, reproductive performance, net reproductive rate, intrinsic and finite rates of population increase on Cry1Ac/Cry1F-soybean and non-*Bt* soybean. In contrast, heterozygotes had their fertility life table parameters significantly reduced on Cry1Ac/Cry1F-soybean. Therefore, the selection of *S. frugiperda* for resistance to single and pyramided *Bt* maize can result in cross-crop resistance to DAS-444Ø6-6 × DAS-81419-2 soybean. The importance of these results to integrated pest management (IPM) and insect resistance management (IRM) programs is discussed.

## Introduction

Transgenic plants expressing insecticidal proteins from *Bacillus thuringiensis* Berliner (*Bt*) have significantly contributed to IPM programs worldwide in the last decades^1–4^. Brazil is one of the largest adopter of biotech crops that express *Bt* proteins in the world, with approximately 36 million hectares of cultivated area during the 2017/2018 season, representing 62, 79 and 82% of the total area planted with soybean, maize and cotton, respectively^4^.

Brazil was also the first country in the world to approve the commercial release of *Bt*-soybean expressing the Cry1Ac protein (event MON87701 × MON89788)^5^, which has been cultivated since 2013/2014 season. This biotech event provided control of important soybean pests, such as *Anticarsia gemmatalis* (Lepidoptera: Erebidae), *Chrysodeixis includens*, *Chloridea virescens* and *Helicoverpa armigera* (Lepidoptera: Noctuidae)^6–10^. Recently, a new *Bt* soybean (event DAS-444Ø6-6 × DAS-81419-2) was approved for commercialization in Brazil^11^. The DAS-444Ø6-6 event (Enlist E3, Corteva Agriscience, Wilmington, DE) expresses the enzymes 5-enolpyruvyl shikimate-3-phosphate synthase (2mEPSPS), phosphinothricin acetyltransferase (PAT), and aryloxyalkanoate dioxygenase 12 (AAD-12) that confer tolerance to the herbicides glyphosate, glufosinate ammonium, and 2,4-dichlorophenoxyacetic acid (2,4-d), respectively. The DAS-81419-2 event (Conkesta, Corteva Agriscience, Wilmington, DE) consists of insect-resistant technology that expresses Cry1Ac and Cry1F *Bt* proteins^11,12^, and PAT that confers tolerance to the herbicide glufosinate ammonium as a selectable marker. Under field conditions, this *Bt* soybean provides protection against *A. gemmatalis, C. includens*, *C. virescens* and *H. armigera*^13,14^.

In Brazilian soybean fields there has been an increase in the occurrence of *Spodoptera* species, mainly *Spodoptera frugiperda* (Lepidoptera: Noctuidae)^15–17^—one the main lepidopteran pest of maize (*Zea ma*ys L.) and cotton (*Gossypium hirsutum* L.)^18,19^. Their occurrence in soybean can be explained by their ability to develop in several cultivated plants^20,21^, adult dispersal^22^, reproductive capacity, multiple generations per year^23^ and the Brazilian crop production system where there is an overlap of cultivated host plants (i.e. maize, cotton, sorghum, rice and soybean)^24^. These biological characteristics associated with the crop production landscapes favors the infestation of this pest on distinct cultivated host plants throughout the seasons.

Field-evolved resistance of *S. frugiperda* to Cry1F^25^ and Cry1Ab^26^ proteins in Brazil resulted in high survival rates on maize and cotton plants expressing pyramided *Bt* proteins^27,28^. Field-evolved resistance to Cry1F maize has also been documented in Puerto Rico^29^, some areas of the southeastern region of the mainland United States^30^, and Argentina^31^. The resistance of *S. frugiperda* to Cry1 proteins expressed in maize negatively affected the performance of *Bt* cotton technologies^32,33^, due to cross-resistance between *Bt* proteins^34–37^. Therefore, the resistance of *S. frugiperda* to *Bt* proteins is the main threat to the sustainability of current and future *Bt* plants used in IPM programs in Brazil.

In the Brazilian crop production landscapes, *S. frugiperda* resistant to *Bt* proteins will also be exposed to DAS-444Ø6-6 × DAS-81419-2 soybean. Therefore, evaluating the ability of *S. frugiperda* strains that have been selected for resistance to *Bt* maize to survive and develop on Cry1Ac/Cry1F-soybean is essential to support IPM and IRM programs. Here, we present data on the survival and development of *S. frugiperda* strains selected for resistance to single and pyramided *Bt* maize, as well as F_1_ hybrids between these and a susceptible strain (assumed to be heterozygous for *Bt* resistance), on DAS-444Ø6-6 × DAS-81419-2 soybeans (hereafter Cry1Ac/Cry1F-soybean).

## Results

### Plant tissue bioassays

Homozygous-resistant *S. frugiperda* from P-R (selected for resistance to Cry1F/Cry1A.105/Cry2Ab2) and H-R (selected for resistance to Cry1F) strains had similar mortality, stunting and larval weights when fed on Cry1Ac/Cry1F-soybean and non-*Bt* soybean (Table 1). In contrast, heterozygous larvae from reciprocal crosses (P-R♀ × Sus♂ and H-R♀ × Sus♂) showed a significant higher mortality on Cry1Ac/Cry1F-soybean (68 and 67%) than on non-*Bt* soybean (< 7% mortality). More than 70% of heterozygous larvae on Cry1Ac/Cry1F-soybean did not reach third instar at five days and larval weights were reduced by more than 50% compared to the same strains on non-*Bt* soybean. The susceptible strain (Sus) had higher mortality (97 and 82%), stunting and weight reduction on Cry1Ac/Cry1F-soybean than on non-*Bt* soybean (2 and 8% mortality and stunting, respectively) (Table 1).

**Table 1:** Percent mortality, stunting (larvae did not reach third instar) and mean weight (mg/larvae) of *S. frugiperda* strains after five days on leaves of Cry1Ac/Cry1F-soybean (event DAS-444Ø6-6 × DAS-81419-2) and non-*Bt* soybean (isoline) in laboratory trials.

*S. frugiperda* strain^a^	% Mortality (95% CI)^b^		% Stunting (95% CI)^b^		Mean weight ± SE^c^
	V_5–6_	R_4–5_	V_5–6_	R_4–5_	R_4–5_
**P-R**					
Cry1Ac/Cry1F-soybean	7.0 (3.0–13.0) a	10.0 (5.0–17.0) a	19.0 (12.0–27.0) a	14.0 (8.0–21.0) a	5.2 ± 0.3 a
Non-*Bt* soybean	7.0 (3.0–13.0) a	2.0 (0.4–6.0) a	14.0 (8.0–21.0) a	5.0 (1.9–10.0) a	5.3 ± 0.2 a
**H-R**					
Cry1Ac/Cry1F-soybean	9.0 (4.0–15.0) a	5.0 (1.9–10.0) a	14.0 (8.0–21.0) a	12.0 (6.0–19.0) a	3.8 ± 0.2 a
Non-*Bt* soybean	3.0 (0.8–8.0) a	3.0 (0.8–8.0) a	9.0 (4.0–15.0) a	8.0 (3.0–14.0) a	3.3 ± 0.1 a
**P-R♀ × Sus♂**					
Cry1Ac/Cry1F-soybean	68.0 (58.4–76.5) a	35.0 (26.2–44.7) a	97.0 (91.7–99.1) a	70.0 (60.5–78.3) a	1.2 ± 0.2 b
Non-*Bt* soybean	2.0 (0.4–6.8) b	5.0 (1.9–10.9) b	3.0 (0.9–8.2) b	11.0 (6.0–18.4) b	7.3 ± 0.8 a
**H-R♀ × Sus♂**					
Cry1Ac/Cry1F-soybean	67.0 (57.3–75.6) a	49.0 (39.3–58.7) a	94.0 (87.8–97.4) a	76.0 (66.9–83.5) a	1.2 ± 0.2 b
Non-*Bt* soybean	1.0 (0.1–5.5) b	7.0 (3.2–13.5) b	3.0 (0.9–8.2) b	12.0 (6.7–19.6) b	2.4 ± 0.3 a
**Sus**					
Cry1Ac/Cry1F-soybean	97.0 (91.7–99.1) a	82.0 (69.5–90.7) a	100.0 (96.4–100.0) a	100.0 (92.9–100.0) a	0.3 ± 0.1 b
Non-*Bt* soybean	2.0 (0.4–6.8) b	2.0 (0.2–10.5) b	8.0 (3.9–14.7) b	3.0 (0.8–8.2) b	5.9 ± 0.6 a

^a^P-R strain (selected for resistance to Cry1F/Cry1A.105/Cry2Ab2-maize), H-R strain (selected for resistance to Cry1F-maize), and Sus strain (susceptible of reference).

^b^Values represent means and respective 95% confidence intervals (CIs) or standard error (SE). For each pair of means, those followed by the same letter in a column and *S. frugiperda* strain are not significantly different due to nonoverlap of 95% CIs.

^c^A separate *t-*test (*P* < 0.05) was conducted between Cry1Ac/Cry1F-soybean and the non-*Bt* soybean for each growth stage and *S. frugiperda* strain (for each pair of means, those followed by different letter in each column and *S. frugiperda* strain are significantly different).

### Life history traits of *S. frugiperda* strains on Cry1Ac/Cry1F-soybean

**Figure 1:**
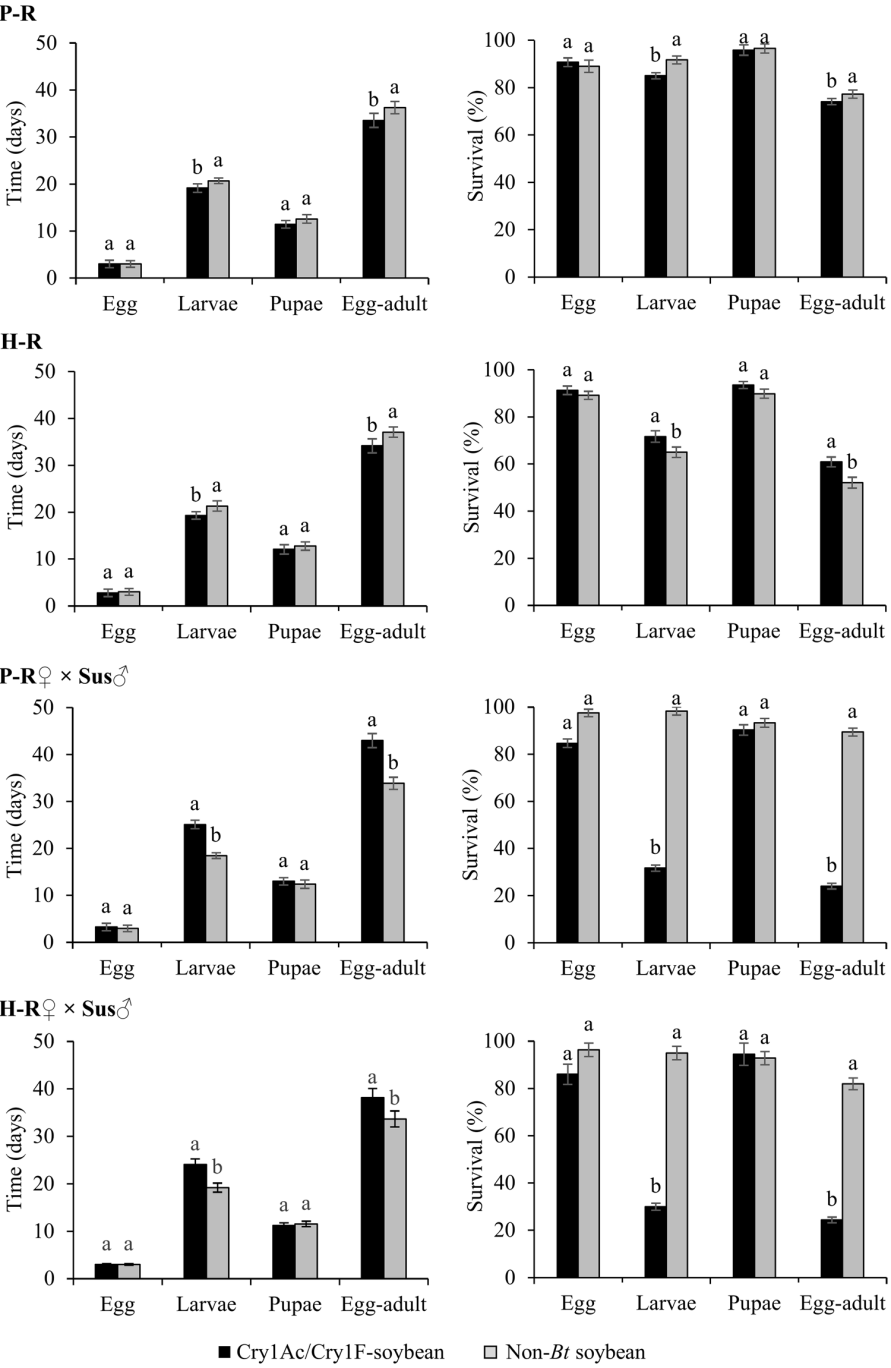
Life history traits of *S. frugiperda* strains on leaves of Cry1Ac/Cry1F-soybean (event DAS-444Ø6-6 × DAS-81419-2) and non-*Bt* soybean (isoline). Pairs of bars (± SE) with different letters differ significantly by *t*-test (*P* < 0.05). P-R strain (selected for resistance to Cry1F/Cry1A.105/Cry2Ab2-maize), H-R strain (selected for resistance to Cry1F-maize), and Sus strain (susceptible of reference).

No significant differences in the duration and survival of egg and pupal stages of homozygous-resistant (P-R and H-R) and heterozygous insects on Cry1Ac/Cry1F-soybean and non-*Bt* soybean were detected (Fig. 1). However, larval stage duration of P-R and H-R strains were significantly shorter (~ 2 days) on Cry1Ac/Cry1F-soybean, and this also reduced the egg-to-adult period when compared to the same strains developing on non-*Bt* soybean. Larval and egg-to-adult survival of P-R strain were significantly lower on Cry1Ac/Cry1F-soybean, while H-R strain showed higher survival on non-*Bt* (Fig. 1). For heterozygotes, the duration of larval (25 and 24 days) and egg-to-adult (43 and 38 days) periods was longer on Cry1Ac/Cry1F-soybean than non-*Bt* (18 and 33 days, respectively) (Fig. 1). However, the survival of heterozygous larvae on Cry1Ac/Cry1F-soybean was lower than 35%, while on non-*Bt* was higher than 95%. There was also a reduction in the number of heterozygous insects that completed the life cycle on Cry1Ac/Cry1F-soybean (24% reaching the adult stage), when compared to non-*Bt* (more than 80% originated adults). In contrast, susceptible insects did not survive until adult stage on Cry1Ac/Cry1F-soybean, while on non-*Bt* more than 60% developed into adults in 35 days. When fed on the same plant (Supplementary Fig. S1), P-R insects had higher survival from neonate to adult on Cry1Ac/Cry1F-soybean (82%) than H-R (67%), heterozygous (28%) and Sus (no survival) insects. On non-*Bt* soybean, the survival of P-R and heterozygous (higher than 88%) insects were similar, while H-R and Sus insects had a lower survival (58% and 75%, respectively). Based on previous results, the dominance levels (*D*_*ML*_) of resistance of *S. frugiperda* on Cry1Ac/Cry1F-soybean were 0.32 (95% CI 0.29–0.36) and 0.40 (95% CI 0.37–0.43) for P-R and H-R strains, respectively, indicating that the resistance is partially recessive.

No significant differences were detected in the larval weights of P-R and H-R insects on Cry1Ac/Cry1F-soybean and non-*Bt* soybean (Table 2). By contrast, pupal weight of resistant insects was significantly heavier on Cry1Ac/Cry1F-soybean than on non-*Bt*. Heterozygous larvae from both resistant strains presented lower weight on Cry1Ac/Cry1F-soybean, but the progeny from P-R♀ × Sus♂ had similar pupal weight on *Bt* and non-*Bt* soybean, while the insects from H-R♀ × Sus♂ had higher pupal weight on non-*Bt*. P-R females produced similar number of eggs when larvae developed on Cry1Ac/Cry1F-soybean or non-*Bt*, while H-R females produced more eggs when their development occurred on *Bt*-soybean. Females from P-R♀ × Sus♂ fed on Cry1Ac/Cry1F-soybean oviposited less eggs than on non-*Bt*. On the other hand, females from H-R♀ × Sus♂ presented similar number of eggs on *Bt* and non-*Bt* soybean. On the same host plant, larval weight was higher for resistant insects on Cry1Ac/Cry1F-soybean than other strains (Supplementary Fig. S2). In contrast, on non-*Bt* soybean heterozygotes had higher larval weight. Pupae were heavier for the H-R insects on Cry1Ac/Cry1F-soybean and H-R♀ × Sus♂ on non-*Bt*. Resistant and heterozygous females on Cry1Ac/Cry1F-soybean produced a similar number of eggs. However, on non-*Bt* heterozygous females produced more eggs.

**Table 2:** Biological parameters of *S. frugiperda* strain on leaves of Cry1Ac/Cry1F-soybean (event DAS-444Ø6-6 × DAS-81419-2) and non-*Bt* soybean (isoline).

Biological parameter^a^	Cry1Ac/Cry1F-soybean^b^	Non-*Bt* soybean^b^	*P-*value
**P-R**			
Larval weight at 14 days (mg)	250.0 ± 9.5	231.2 ± 20.2	0.4349
Pupae weight (mg)	154.8 ± 2.8	141.0 ± 4.2	0.0204
Mean eggs/female	742.0 ± 73.3	916.5 ± 75.3	0.1089
**H-R**			
Larval weight at 14 days (mg)	233.3 ± 19.3	202.4 ± 20.2	0.2942
Pupae weight (mg)	187.5 ± 4.2	157.0 ± 3.9	0.0003
Mean eggs/female	987.7 ± 120.4	636.5 ± 51.2	0.0143
**P-R♀ × Sus♂**			
Larval weight at 14 days (mg)	72.7 ± 12.2	382.1 ± 17.9	< 0.0001
Pupae weight (mg)	169.3 ± 8.2	157.8 ± 9.8	0.1940
Mean eggs/female	859.3 ± 121.0	1,318.5 ± 53.8	0.0007
**H-R♀ × Sus♂**			
Larval weight at 14 days (mg)	73.2 ± 11.8	355.1 ± 12.9	< 0.0001
Pupae weight (mg)	146.1 ± 4.3	180.8 ± 4.0	0.0002
Mean eggs/female	953.6 ± 145.4	1511.6 ± 74.8	0.0011

^a^P-R strain (selected for resistance to Cry1F/Cry1A.105/Cry2Ab2-maize), H-R strain (selected for resistance to Cry1F-maize), and Sus strain (susceptible of reference).

^b^Values represent means ± SE. A separate *t*-test (*P* < 0.05) was conducted between Cry1Ac/Cry1F-soybean and the non-*Bt* soybean for each biological parameter.

Fertility life table parameters of P-R strain on the mean generation time, net reproductive rate, intrinsic and finite rate of population increase was similar on Cry1Ac/Cry1F-soybean and non-*Bt* (Table 3). Based on this, after ~ 40 days, 257 and 326 females from each P-R female are expected on Cry1Ac/Cry1F-soybean and non-*Bt*, respectively. However, H-R females presented higher fertility life table parameters on *Bt* soybean. For this strain, after ~ 40 days, 297 females/female are expected when feeding on Cry1Ac/Cry1F-soybean, while on non-*Bt* only 157 females/female in 45 days. By contrast, heterozygotes on Cry1Ac/Cry1F-soybean had their life history parameters negatively affected. For these insects, 94 and 105 females are expected from each female on Cry1Ac/Cry1F-soybean in 44 to 49 days, while on non-*Bt* soybean more than 540 females/female would be produced in 39 days. This represents a reduction of 80% in the number of females produced per generation on Cry1Ac/Cry1F-soybean. When fertility life table parameters were compared in a same host plant, the homozygous-resistant insects on Cry1Ac/Cry1F-soybean presented shortest generation time, better reproductive performance and rate of population increase (Supplementary Table S1).

**Table 3:** Fertility life table parameters of *S. frugiperda* strains on leaves of Cry1Ac/Cry1F-soybean (event DAS-444Ø6-6 × DAS-81419-2) and non-*Bt* soybean (isoline).

*S. frugiperda* strain^a^	Fertility life table parameter^b,c^			
	*T* (days)	*R*_*o*_ (♀ / ♀)	*r*_*m*_ (♀/♀*day)	*λ*
**P-R**				
Cry1Ac/Cry1F-soybean	39.37 ± 0.12 a	257.80 ± 24.80 a	0.14 ± 0.003 a	1.15 ± 0.003 a
Non-*Bt* soybean	40.55 ± 0.29 a	326.45 ± 27.78 a	0.14 ± 0.004 a	1.15 ± 0.002 a
**H-R**				
Cry1Ac/Cry1F-soybean	40.59 ± 0.51 b	297.97 ± 37.07 a	0.14 ± 0.003 a	1.15 ± 0.003 a
Non-*Bt* soybean	45.80 ± 0.36 a	156.98 ± 12.56 b	0.11 ± 0.002 b	1.12 ± 0.003 b
**P-R♀ × Sus♂**				
Cry1Ac/Cry1F-soybean	49.50 ± 0.29 a	94.87 ± 13.36 b	0.09 ± 0.003 b	1.09 ± 0.003 b
Non-*Bt* soybean	39.77 ± 0.13 b	539.79 ± 22.04 a	0.16 ± 0.001 a	1.17 ± 0.001 a
**H-R♀ × Sus♂**				
Cry1Ac/Cry1F-soybean	44.83 ± 0.41 a	105.27 ± 16.04 b	0.10 ± 0.003 b	1.11 ± 0.003 b
Non-*Bt* soybean	39.81 ± 0.14 b	570.16 ± 28.21 a	0.16 ± 0.002 a	1.17 ± 0.001 a

^a^P-R strain (selected for resistance to Cry1F/Cry1A.105/Cry2Ab2-maize), H-R strain (selected for resistance to Cry1F-maize), and Sus strain (susceptible of reference).

^b^*T* = mean length of a generation (days); *R*_*o*_ = net reproductive rate (females per female per generation); *r*_*m*_= intrinsic rate of population increase (per day); = λ finite rate of population increase (per day).

^c^Means within a column followed by the same letter in each *S. frugiperda* strain are not significantly different (*t*-tests for pairwise group comparisons, *P* > 0.05).

## Discussion

The colonies of *S. frugiperda* selected for resistance to single- and pyramided-*Bt* maize technologies showed high survival on Cry1Ac/Cry1F-soybean (event DAS-444Ø6-6 × DAS-81419-2). While quantitative measurements of Cry1Ac/Cry1F *Bt* protein expression were not collected in our study, observations from previous studies have shown that Cry1Ac/Cry1F protein expression in greenhouse-grown plants does fall within the range of expression observed across field environments (Corteva Agriscience, unpublished data). Measurements of *Bt* protein expression can vary across environments and be influenced by multiple factors, such as growth stage, position within the plant canopy, and type of plant tissue. The amount of Cry1Ac and Cry1F protein expressed in this soybean technology was reported in the USDA petition for nonregulated status^38^ and by De Cerqueira et al.^39^. Therefore, the high survival of resistant strains on Cry1Ac/Cry1F-soybean can be explained by the cross-resistance between Cry1 proteins expressed in *Bt* plants^34–37^ and their low natural susceptibility to Cry1Ac protein as reported in studies with Cry1Ac-cotton^32,40–42^, Cry1Ac-soybean^43,44^, and diet bioassays containing Cry1Ac^43,45^. The cross-resistance among Cry1 proteins is attributed to their similar amino acid sequence^36^ and also their same binding sites in the midgut of *S. frugiperda*^35^. Previous studies also showed that *S. frugiperda* resistant to *Bt* maize survived on single and pyramided *Bt* cotton^32,33^, indicating cross-crop resistance. Our results also revealed that homozygous-resistant insects had similar development and reproductive performance on Cry1Ac/Cry1F-soybean and non-*Bt* soybean. These finding indicate that resistant insects have no adaptive disadvantage in the absence of the selection agent, maintaining the resistance frequency in the field^46^.

By contrast, heterozygous insects showed lower survival than homozygous-resistant insects on Cry1Ac/Cry1F-soybean, but produced fertile adults. The survival of heterozygous insects on Cry1Ac/Cry1F-soybean indicated that the dominance levels of resistance is characterized as partially recessive for both resistant strains evaluated. In other words, it demonstrates that the Cry1Ac/Cry1F-soybean does not meet the high-dose definition (*Bt* protein expression that cause more than 95% mortality of heterozygotes)^47^ for *S. frugiperda*. A similar degree of resistance was reported in *S. frugiperda* strains selected for resistance to *Bt* maize when fed on leaf tissue of Cry1F-maize^29,31,48^, and cotton events expressing Cry1Ac/Cry1F, Cry1Ac/Cry2Ab2, and Cry1Ab/Cry2Ae *Bt* proteins^32,33^. The survival of heterozygous larvae on Cry1Ac/Cry1F-soybean also contributes to maintaining the resistance allele to Cry1 proteins in field populations. On the other hand, heterozygotes on Cry1Ac/Cry1F-soybean had lower larval weight and longer development time until adults. This feature could be exploited in IPM programs by increasing the exposure on the plant to beneficial arthropods or entomopathogenic agents. Unlike to previous results, the susceptible *S. frugiperda* had complete mortality on Cry1Ac/Cry1F-soybean, due to its high susceptibility to Cry1F protein, as previous reported before the field-evolved resistance of this species to Cry1F-maize^25,27–34^.

In the current Brazilian crop production landscapes, with successive cultivation of maize, cotton and soybean, *S. frugiperda* populations are exposed to high selection pressure for resistance to *Bt* proteins. Resistance has been observed in *S. frugiperda* field populations to several *Bt* proteins expressed in maize (i.e. Cry1F, Cry1Ab and Cry1A.105) in Brazil^25–27^. Currently, field populations of *S. frugiperda* are composed predominantly of insects carrying Cry1Ab, Cry1F, and Cry1A.105 resistance alleles^26,28,49–51^, reflected by the increasing use of insecticide applications in fields cultivated with crops expressing these proteins^52^. For example, on single or pyramided *Bt* maize technologies expressing Cry1 and Cry2 proteins up to four insecticidal sprays may be needed to manage *S. frugiperda*, under extreme infestations^52,53^. Based on this, it is expected that Cry1Ac/Cry1F-soybean may not provide stand-alone protection against *S. frugiperda* under Brazilian field conditions, making this species a non-target pest of this *Bt* technology. However, Cry1Ac/Cry1F-soybean was developed and does provide efficacy against the key soybean pests (*A. gemmatalis*, *C. includens*, and *C. virescens*) under field conditions in Brazil^13,14^, which are the driver pests for the development of *Bt* traits in soybean. Therefore, to maintain the effectiveness of Cry1Ac/Cry1F-soybean over time against the key soybean pests, the adoption of a structured refuge (20% of cultivated area should be planted with non-*Bt* soybean) will be important for delaying or preventing resistance evolution^33,36,47^.

According to our results, alternative IPM strategies will be necessary to control *S. frugiperda* on Cry1Ac/Cry1F-soybean. Therefore, monitoring the presence of larvae and the damage to Cry1Ac/Cry1F-soybean are essential for supporting decision making regarding the use of other IPM tactics. The use of chemical insecticides probably will be the main tactic against *S. frugiperda* on *Bt* soybean. However, Cry1Ac/Cry1F-soybean could also be integrated with biological control agents as baculovirus-based insecticides (e.g. *Spodoptera frugiperda* multiple nucleopolyhedrovirus—SfMNPV)^54^ and natural enemies^55^. In summary, IPM and IRM programs that integrate multiple control tactics with diverse mortality factors, rather than just relying on wide scale use of single control tactics like *Bt* crops, are needed to ensure the sustainability of *Bt* crops^56^ in Brazil, where the resistance of *S. frugiperda* to Cry1 *Bt* proteins is already widespread.

## Methods

### Description of *S. frugiperda* strains

Two putative *S. frugiperda* resistant colonies were selected from a field population collected in maize in Paulínia, São Paulo, Brazil (22° 42′ 38′′ S and 47° 06′ 26′′ W) using the F_2_ screen method developed by Andow and Alstad^57^. The selection and rearing of resistant colonies was described in detail by Muraro et al.^53^. The homozygous-resistant strains used in this study were H-R (selected for resistance to Cry1F-maize) and P-R (selected for resistance to Cry1F/Cry1A.105/Cry2Ab2-maize). We also used a strain of *S. frugiperda* that has been maintained in the laboratory since 2012 without exposure to *Bt* proteins. This population was collected in non-*Bt* maize during the 2011–2012 crop season in Mogi Mirim, São Paulo, Brazil (22° 28′ 31′′ S and 46° 54′ 21′′ W). We refer to this colony as a susceptible strain (Sus). To evaluate putative heterozygous insects, the crossing between resistant♀ × susceptible♂ were performed. We only used heterozygotes from this cross because inheritance of resistance is autosomal inherited, and heterozygous larvae have demonstrated similar mortality-response to *Bt* proteins in diet and leaf bioassays^25,27–29,33,53^.

### Soybean plants

Seeds from Cry1Ac/Cry1F-soybean and non-*Bt* soybean (isoline) (maturity group 5.0) were sown in 12-l plastic pots (four seeds/pot) in a greenhouse. Before the bioassays, *Bt* and non-*Bt* plants were tested for *Bt* protein expression using detection kits for Cry1Ac and Cry1F (Envirologix, QuickStix).

### Plant tissue bioassays

Bioassays were performed with soybean leaves from Cry1Ac/Cry1F-soybean and non-*Bt* soybean (isoline) in V_5–6_ and R_4–5_ growth stages. Leaves were removed from the upper third part of the plants and, in the laboratory, were placed on a gelled mixture of agar-water at 2.5% in 100 ml plastic cups. Subsequently, neonates from resistant or susceptible strains or their F_1_ hybrid were placed on each cup. Cups were sealed and maintained in a room at 25 ± 2 °C, 60 ± 10% RH, and a photophase of 14 h. The experimental design was completely randomized with 10 replicates of 10 neonates/strain/growth stage. Mortality, stunting (larvae that did not reach the 3rd instar) and weight were assessed after 5 days.

### Life history traits of *S. frugiperda* strains on Cry1Ac/Cry1F-soybean

Homozygous-resistant, heterozygous or susceptible neonates were reared on leaves of Cry1Ac/Cry1F-soybean and non-*Bt* soybean (isoline) excised from greenhouse-grown plants at the R_1_ growth stage. In the laboratory, leaves were cut into pieces and placed on a gelled mixture of agar-water at 2.5% in 50 ml plastic cups. Then, a single neonate (6 replicates of 10 neonates/strain/treatment) was placed in each cup. Leaves were replaced every 48 h and cups were maintained in the same environmental conditions described above. The following life history traits were evaluated: duration and survival of egg, larva, pupa and total cycle periods (egg-to-adult); larval weight at 14 days; pupae weight 24 h after pupal formation; and number of eggs per female. The number of eggs were assessed daily from 18 couples kept in PVC cages (23-cm height × 10-cm diameter) internally coated with a paper towel and closed at the top with a voile-type fabric. To determine the embryonic period and survival, 100 eggs of the 2nd oviposition were obtained from each couple. The eggs were observed daily and the number of hatched larvae was counted.

### Data analysis

The number of insects tested, dead and those did not develop to 3rd instar on Cry1Ac/Cry1F-soybean and non-*Bt* soybean (isoline) were used to estimate 95% confidence intervals (CIs) for the probability of mortality and stunting, according to a binomial distribution. For these analyses, the function *binom.probit* from the package *binom* in R 3.1.0 (R Development Core Team 2014)^58,59^ was used. Percent mortality and stunting were considered significantly different when the 95% CIs on *Bt*-soybean did not overlap the 95% CIs on non-*Bt* soybean. The life history data of *S. frugiperda* strains on *Bt* and non-*Bt* soybean met the assumptions of normality and homogeneity of variances, and were compared by *t-*test using the PROC TTEST procedure in SAS 9.1^60^. Mortality until adult stage were used to estimate the effective dominance of resistance (*D*_*ML*_) using the method described by Bourguet et al.^61^ based on the following equation: *D*_*ML*_ = (*M*_*RS*_ – *M*_*SS*_)*/*(*M*_*RR*_ – *M*_*SS*_), where *M*_*SS*_, *M*_*RR*_ and *M*_*RS*_ are the mortalities of the susceptible, resistant and heterozygous strains, respectively, on *Bt*-soybean. This equation estimates dominance levels on a scale of 0 to 1 (0 = complete recessivity and 1 = complete dominance). The 95% CIs for these estimates was calculated as proposed by Misra^62^. A fertility life table was also calculated by estimating the mean generation time (*T*), the net reproductive rate (*R*_*o*_), and the intrinsic (*r*_*m*_) and finite (*λ*) rate of increases by the jackknife technique using “*lifetable.sas*” procedure developed by Maia et al.^63^ in SAS 9.1^60^.

## Supplementary information

Supplementary information
