# Supplementary material for: Cross-crop resistance of Spodoptera frugiperda selected on Bt maize to genetically-modified soybean expressing Cry1Ac and Cry1F proteins in Brazil
Source: Sci Rep. 2020 Jun 22;10:10080. doi: 10.1038/s41598-020-67339-1 (PMC7308303; doi:10.1038/s41598-020-67339-1)
Supplement: Supplementary file 1 — Supplementary information [file 41598_2020_67339_MOESM1_ESM.docx]

**Cross-crop resistance of *Spodoptera frugiperda* selected on *Bt* maize to genetically-modified soybean expressing Cry1Ac and Cry1F proteins in Brazil**

Eduardo P. Machado^1^, Gerson L. dos S. Rodrigues Junior^1^, Fábio M. Führ^1^, Stefan L. Zago^1^, Luiz H. Marques^2^, Antonio C. Santos^2^, Timothy Nowatzki^3^, Mark L. Dahmer^3^, Celso Omoto^4^, Oderlei Bernardi^1*^

^1^Department of Plant Protection, Federal University of Santa Maria (UFSM), Roraima avenue 1000, Santa Maria 97105-900, Rio Grande do Sul, Brazil.

^2^Corteva Agriscience, Alameda Itapecuru, 506, Alphaville, Barueri - SP, 06454-080, Brazil.

^3^Corteva Agriscience, 7000NW 62nd Ave, Johnston, IA 50131, USA.

^4^Department of Entomology and Acarology, Luiz de Queiroz College of Agriculture (ESALQ), University of São Paulo (USP), Pádua Dias avenue 11, Piracicaba 13418-900, São Paulo, Brazil.

*Correspondence and requests for materials should be addressed to OB (e-mail: oderlei.bernardi@ufsm.br) or LHM (luiz.marques@corteva.com)


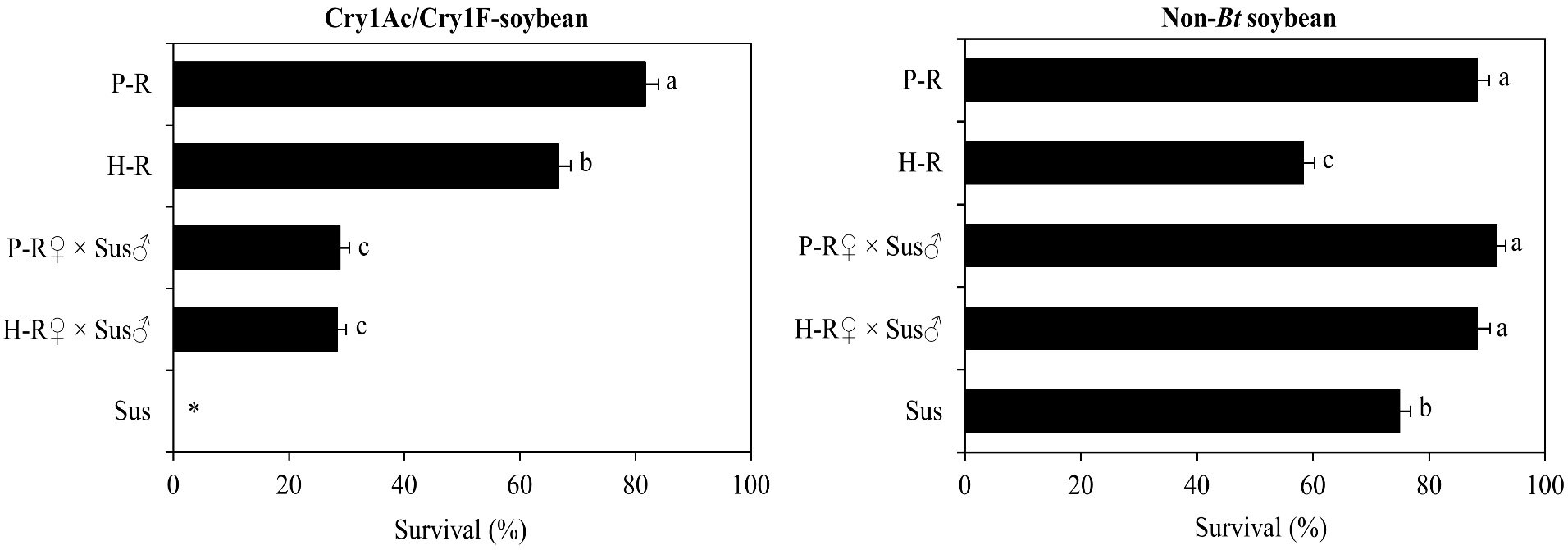


**Supplementary Figure S1.** Survival from neonate to adult of *S. frugiperda* strain on leaves of Cry1Ac/Cry1F-soybean (event DAS-444Ø6-6 × DAS-81419-2) and non-*Bt* soybean (isoline). Bars (± SE) with different letters differ significantly by *t*-test (*P* < 0.05). P-R strain (selected for resistance to Cry1F/Cry1A.105/Cry2Ab2-maize), H-R strain (selected for resistance to Cry1F-maize), and Sus strain (susceptible of reference). An asterisk (*) indicates that standard errors were not estimated because no variability existed.


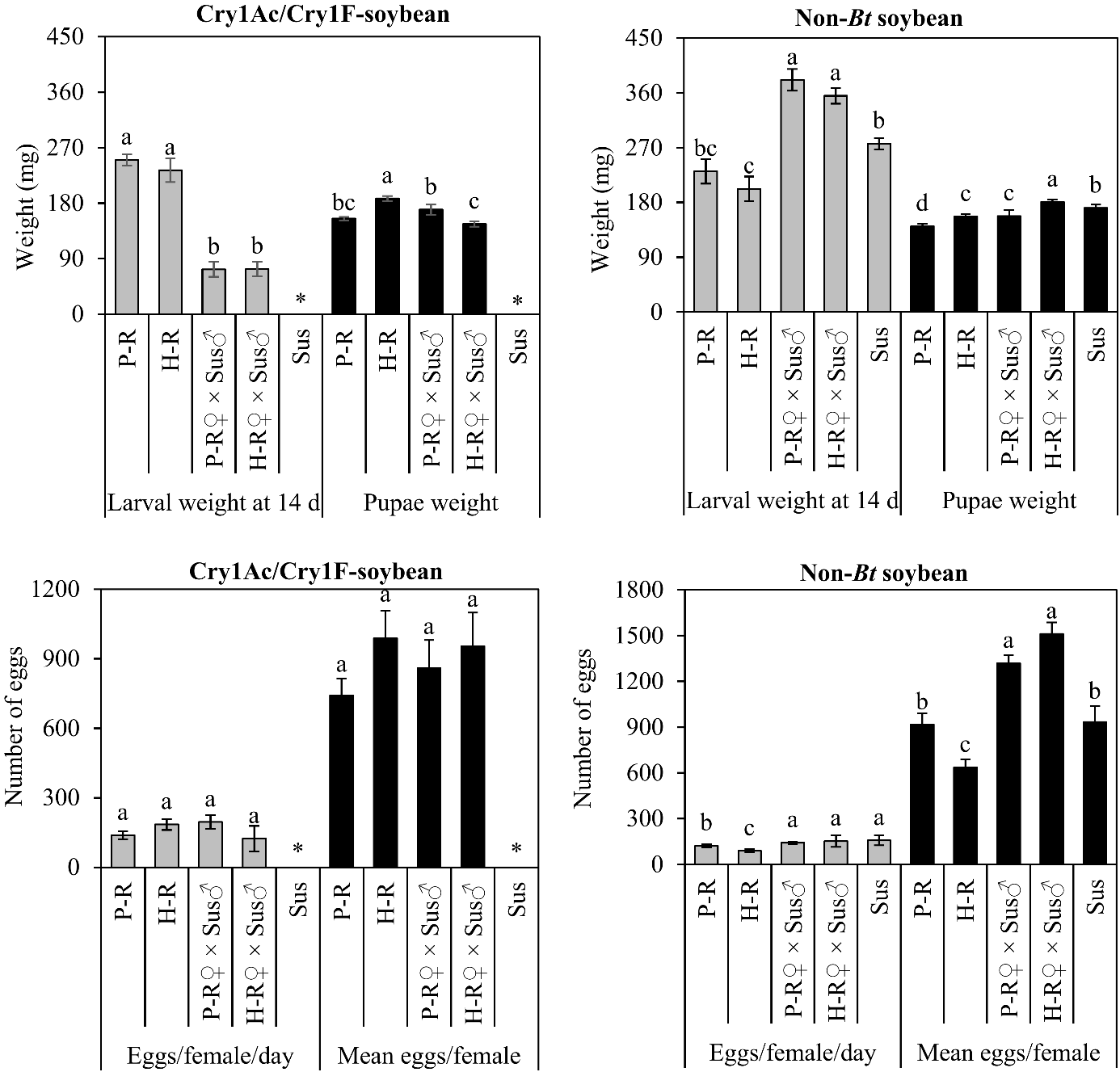


**Supplementary Figure S2.** Average weight of larvae, pupae and number of eggs of *S. frugiperda* strains on leaves of Cry1Ac/Cry1F-soybean (event DAS-444Ø6-6 × DAS-81419-2) and non-*Bt* soybean (isoline). Bars (± SE) with different letters for each variable differ significantly by *t*-test (*P* < 0.05). P-R strain (selected for resistance to Cry1F/Cry1A.105/Cry2Ab2-maize), H-R strain (selected for resistance to Cry1F-maize), and Sus strain (susceptible of reference). An asterisk (*) indicates that standard errors were not estimated because no variability existed.

**Supplementary Table S1.** Life table parameters of *S. frugiperda* strains on leaves of Cry1Ac/Cry1F-soybean (event DAS-444Ø6-6 × DAS-81419-2) and non-*Bt* soybean (isoline).

| *S. frugiperda* strain^a^ | Fertility life table parameter^b,c^ | | | | |
| --- | --- | --- | --- | --- | --- |
|  | *T* (days) | *R_o_* (♀ / ♀) | *r_m_* (♀ / ♀*day) | *λ* |  |
| **Cry1Ac/Cry1F-soybean** | | | | | |
| P-R | 39.37 ± 0.12 c | 257.80 ± 24.80 a | 0.14 ± 0.003 a | 1.15 ± 0.003 a |  |
| H-R | 40.59 ± 0.51 c | 297.97 ± 37.07 a | 0.14 ± 0.003 a | 1.15 ± 0.003 a |  |
| P-R♀ × Sus♂ | 49.50 ± 0.29 a | 94.87 ± 13.36 b | 0.09 ± 0.003 b | 1.09 ± 0.003 b |  |
| H-R♀ × Sus♂ | 44.83 ± 0.41 b | 105.27 ± 16.04 b | 0.10 ± 0.003 b | 1.11 ± 0.003 b |  |
| Sus | –^c^ | – | – | – |  |
| **Non-*Bt* soybean** |  |  |  |  |  |
| P-R | 40.55 ± 0.29 c | 326.45 ± 27.78 b | 0.14 ± 0.004 b | 1.15 ± 0.002 b |  |
| H-R | 45.80 ± 0.36 a | 156.98 ± 12.56 c | 0.11 ± 0.002 c | 1.12 ± 0.003 c |  |
| P-R♀ × Sus♂ | 39.77 ± 0.13 c | 539.79 ± 22.04 a | 0.16 ± 0.001 a | 1.17 ± 0.001 a |  |
| H-R♀ × Sus♂ | 39.81 ± 0.14 c | 570.16 ± 28.21 a | 0.16 ± 0.002 a | 1.17 ± 0.001 a |  |
| Sus | 42.90 ± 0.39 b | 277.84 ± 13.80 b | 0.13 ± 0.002 b | 1.14 ± 0.002 b |  |

a) P-R strain (selected for resistance to Cry1F/Cry1A.105/Cry2Ab2-maize), H-R strain (selected for resistance to Cry1F-maize), and Sus strain (susceptible of reference). b) *T* = mean length of a generation (days); *R_o_* = net reproductive rate (females per female per generation); *r_m_* = intrinsic rate of population increase (per day); *λ* = finite rate of population increase (per day). c) Means within a column followed by the same letter in each host plant are not significantly different (*t*-tests for pairwise group comparisons, *P* > 0.05). c) There is no survival insects of Sus strain on Cry1Ac/Cry1F-soybean.
